# A Randomized Controlled Trail Comparing the Efficacy of 0.5% Centbucridine to 2% Lignocaine as Local Anesthetics in Dental Extractions

**DOI:** 10.1155/2011/795047

**Published:** 2011-11-22

**Authors:** Samir Mansuri, Ahmed Bhayat, Esam Omar, Fadi Jarab, Mohammad Sami Ahmed

**Affiliations:** ^1^Oral & Maxillofacial Surgery, College of Dentistry, Taibah University, Hizaam Street, P.O. Box 1263, Madinah Munawarah, Saudi Arabia; ^2^Dental Public Health, College of Dentistry, Taibah University, Hizaam Street, P.O. Box 1263, Madinah Munawarah, Saudi Arabia

## Abstract

The development of local anesthesia in dentistry has marked the beginning of a new era in terms of pain control. Lignocaine is the most commonly used local anesthetic (LA) agent even though it has a vasodilative effect and needs to be combined with adrenaline. Centbucridine is a non-ester, non amide group LA and has not been comprehensively studied in the dental setting and the objective was to compare it to Lignocaine. This was a randomized study comparing the onset time, duration, depth and cardiovascular parameters between Centbucridine (0.5%) and Lignocaine (2%). The study was conducted in the dental outpatient department at the Government Dental College in India on patients attending for the extraction of lower molars. A total of 198 patients were included and there were no significant differences between the LAs except those who received Centbucridine reported a significantly longer duration of anesthesia compared to those who received Lignocaine. None of the patients reported any side effects. Centbucridine was well tolerated and its substantial duration of anesthesia could be attributed to its chemical compound. Centbucridine can be used for dental procedures and can confidently be used in patients who cannot tolerate Lignocaine or where adrenaline is contraindicated.

## 1. Introduction

The ability to provide the patient with clinically adequate pain control is one of the major concerns all over the world. The development of local anesthesia has marked the beginning of new era in the field of dentistry. The use of local anesthetics (LAs) in dentistry and other surgical procedures as a means of pain control has been one of the medical marvels of twentieth century [1, 2]. Today Lignocaine is the most commonly used local anesthetic agent in dentistry and is referred to as the “gold standard” for dental procedures [1, 2]. Although its properties resemble an ideal LA agent, it is not completely free from cardiovascular toxicity and has an inherent vasodilating property [1, 3, 4]. As a result of the vasodilating characteristic, it has to be combined with a vasoconstrictor, such as adrenaline, to decrease its rate of absorption at the injection site and hence prolong the duration and depth of anesthesia. The use of adrenaline as a vasoconstrictor is sometimes contraindicated for medically compromised patients. To overcome these disadvantages, other LAs have been developed over the past few years, including Centbucridine.

Centbucridine, chemically known as 4-N-butylamino-1,2,3,4-tetrahydroacridine hydrochloride, is a new quinoline derivative with LA action. It was synthesized at the central drug research institute, Lucknow, India, by Patnaik et al. and has the advantage of having an inherent vasoconstrictor property [4].

Many studies have been done on Centbucridine in the medical field including ophthalmic surgery [5, 6], subarachnoid [7], and spinal [8] blocks. However, in dentistry, only a few have been published [2, 9, 10], and most of them were carried out in the early eighties. However, all of the published literature has shown Centbucridine as a potent and reversible LA. One study reported it to be four to five times more potent than Lignocaine [7]. It produced fewer side effects, better cardiovascular stability, and no sensitivity reactions. It has been proven to be as effective as other LA agents in ophthalmology [5, 6] and in subarachnoid blocks [7]. It has not been thoroughly evaluated in the dental field, and therefore this study was done to evaluate its efficacy.

The aim was to compare the efficacy of 0.5% Centbucridine HCl to 2% Lignocaine HCl with Adrenaline (1 : 200 000) for various parameters required in the dental field.

## 2. Objectives

1. To assess and compare the onset (in seconds), duration (in minutes), and depth (using a visual analogue sore) of anesthesia in healthy adults between Lignocaine and Centbucridine.
2. To monitor and compare the cardiovascular response (pulse and blood pressure) in patients on the two LAs.
3. To identify any side effects/allergic reactions to Centbucridine.

## 3. Materials Method

This was a double blind randomized control trial comparing the effects of 2% Lignocaine HCI with adrenaline (1 : 200 000) and 0.5% Centbucridine HCI. It was done during June 2009 till December 2009 on outpatients attending the Gujarat University, Government Dental College in India. All patients were healthy adults according to ASA-I classification and aged between 18 and 60 years old. All patients who attended as outpatients for dental extractions and met the criteria were asked to participate in the study. A total of 198 patients were included in the study. All of the patients attended for the extraction of lower molars. They each randomly received a single anesthetic dose of either 0.5% Centbucridine HCI or 2% Lignocaine HCI with adrenaline. They were unaware of which type of LA they received. Each patient was asked to select one of two brown sealed envelopes which contained the two treatment options. 

The operator administered a single cartridge of LA agent using the Inferior Alveolar Nerve block standardized technique as described in Handbook of Local Anesthesia [1]. The operator was handed the loaded syringe by a qualified medical nurse and was unaware of the type of LA that he was administering. The name of the anesthetic ampoule was covered with a permanent marker which ensured that the operator was blinded. 

The study population was divided into two groups: Group I (Centbucridine) and Group II (Lignocaine). Patients with acute oral infections were excluded. Intradermal sensitivity tests were done prior to administration of the LA, and any patient who tested positive was excluded from the study.

The rationale for the study including its objectives was explained to each participant. Informed and written consent was obtained from all patients. Ethics for the study was obtained from the University Ethical Committee.

The onset, duration, depth, and cardiovascular measurements were carried out by a qualified medical nurse. The time taken for the onset and duration of local anesthesia was measured using a stopwatch.

## 4. Criteria for Assessment

### 4.1. Onset of Anesthesia (Measured in Seconds)

This was measured both objectively and subjectively by the patient in seconds. Anesthesia was confirmed objectively by a pinprick test using a 20 gauge sterile needle which was applied over the attached gingival of the tooth to be extracted. It was confirmed subjectively when the patient first described symptoms of anesthesia for example—numbness or tingling sensation over lower lip. A qualified medical nurse measured the onset using a stopwatch.

### 4.2. Duration of Anesthesia (Measured in Minutes)

This was the time interval between the onset of anesthesia and when the patient reported subjective feelings of normal sensation. This was confirmed objectively by the pinprick test as described above. A qualified medical nurse measured the onset using a stopwatch.

### 4.3. Depth of Anesthesia

This was judged subjectively by the patient using a standardized visual analogue score (VAS). The score ranged from “0” to “5” with “0” being “no pain” and “5” being the most severe intense pain, which the patient could not bear. Each patient was asked to score the “amount” of pain he/she felt during the extraction of the tooth. A low score (0) meant that the patient felt no pain at all; a moderate score (1 and 2) meant that the patient felt mild pain; a score of (3 and 4) meant that the patient felt moderate pain; a high score (5) meant that the patient felt excruciating and unbearable pain.

### 4.4. To Monitor and Compare CVS Response Using Blood Pressure and Heart Rate

The systolic and diastolic blood pressure (BP) was measured in mm of mercury, and the pulse rate was measured using beats per minute. The measurements were done preoperatively (base line), and then at 10, 20, 30, and 60 minute intervals after the administration of the LA. All patients were seated and in the resting position when the measurements were recorded. The same sphygmomanometer was used for all patients, and the nurse completed the recordings using standard guidelines.

### 4.5. Side Effects/Allergic Reaction

Any signs of an allergic reaction including itching, redness, and localized swelling were recorded. 

In cases where additional doses of LA were required, it was recorded separately.

Statistical analysis was done using the student's *t* test with SPSS software. The onset of action and duration of anesthesia was compared between the two groups. Changes in the heart rate (pulse), systolic, and diastolic blood pressure at various time intervals were analyzed against preoperative values in each group. All statistical results less than *P* < 0.05 were considered to be statistically significant.

## 5. Results

There was a fairly equitable distribution of cases between the two groups in terms of age, gender, and baseline blood pressure and pulse readings. A total of 198 patients met the criteria and were included in the study. Of these, 100 patients received Centbucridine (Group I) and 98 received Lignocaine (Group II). There were slightly more females (53%) than males, and the average age was 37.7 years (see Table 1). 

The average time for the onset (seconds) and duration (minutes) of anesthesia is reported in Table 2. Patients who received Centbucridine reported a significantly longer duration of anesthesia compared to those who received Lignocaine. 

Table 3 shows the depth of anesthesia as recorded by the patient using the Visual Analogue Score (VAS). The range was from 0 (no pain) to 5 (severe, unbearable pain). No score more than 1 was noted. No significant differences were found between the two LAs. 

The mean heart rate (beats per minute) at different time intervals is shown below (Table 4). Both LAs significantly increased the heart rate at the 10 minutes interval. At the other later time intervals, there was no significant increase in the mean heart rate. 

In both groups, there was a statistically significant increase in systolic BP at the 10 minute interval, after which there were no significant changes. In terms of the mean diastolic BP, there were no statistically significant differences in the BP over the time period intervals.

No patients required additional doses of LA solutions.

## 6. Discussion

There were almost equal numbers of patients in both groups, and the age and gender distribution was equally distributed. There were no significant differences between the groups in terms of demographical characteristics, and hence the groups could be compared to each other. The average time required for onset of anesthesia was just under three minutes, and Centbucridine was significantly shorter. On average, patients felt the anesthetic effect of Centbucridine about 14 seconds quicker than that of Lignocaine which is clinically not significant. These results are within the reported range of initiation of anesthesia as reported by others to be between 1 and 6 minutes [2, 9]. This could be due to the inherent vasoconstrictive effect of Centbucridine as compared to Lignocaine.

The mean duration of anesthesia was significantly higher for Centbucridine compared to Lignocaine. Patients reported an average anesthesia of 2.5 hours (151 minutes) for Centbucridine compared to under 2 hours (111 minutes) for Lignocaine. A possible reason could be the fact that since Centbucridine has a natural vasoconstrictive effect, the LA solution remained close to and around the nerve tissue for a longer period of time. The solution was prevented from being absorbed and dispersed, and this could have resulted in the longer duration of anesthetic time that was obtained.

 Both LAs showed similar results in terms of depth of anesthesia. No patients reported a score of more than 1 (mild annoying pain), and all patients were sufficiently anesthetized to carry out the procedures. This was similar to other studies [8]. 

There was mild and transient elevation of heart rate in both the groups at the 10 minute interval. However, at all subsequent evaluations, the heart rate had returned to the preanesthesia value. In all of the cases, the tooth was extracted and treatment was complete within 10 minutes. Therefore, after the 10 minute interval, the patients were much more relaxed, the fear had decreased, and their anxiety had been reduced. It was therefore understandable that their heart rate was high at the 10 minute mark but reduced and returned to normal by the 20 minute interval and at all subsequent evaluations. This has been also reported in other studies [2] and considered normal. There was no difference between the blood pressure parameters of both the LAs. Mild elevation of this parameter during initial time was attributed due to anxiety and fear as discussed above. This has also been reported by other authors [1, 2, 9]. Lignocaine has an inherent vasodilating property, which in turn requires adrenaline. This has been shown to increase the blood pressure and heart rate in some studies [1, 9] and is contraindicated to some medically compromised patients. In this study, although there were no medically compromised patients, Lignocaine did not significantly increase the blood pressures and heart rate.

There were no adverse or allergic reactions to either of the LAs in our sample population. Earlier episodes of an allergy to Lignocaine have been reported but are very rare [10–12]. Since the sample population in the study consisted of about 200 patients, it is not surprising that there were no patients who reported adverse reactions. Centbucridine has showed an antihistaminic activity by blocking the H1 histamine receptors which makes it an ideal LA agent in patients with known allergy to other conventional LAs [2, 5]. However, it must be noted that the sample population in the study was relatively small and as a result.

## 7. Conclusion

It can be concluded that Centbucridine produced a significantly longer duration of anesthesia. It worked just as effectively as the “gold standard” Lignocaine, matching it in terms of time of onset, depth of anesthesia, and cardiovascular effects. It produced no side effects or toxic reactions and confirmed its safety for use in this sample population. We can recommend Centbucridine as a LA agent for dental procedures which may last up to 2 hours. It is also recommended that Centbucridine could be confidently used in medically compromised patients where Lignocaine or adrenaline is contraindicated.

## Figures and Tables

**Table 1:** Age and gender distribution.

Group	Mean age	Male	Female	Total
I	38.1	45 (45%)	55 (55%)	100
II	37.2	49 (50%)	49 (50%)	98

Total	37.7	94 (47%)	104 (53%)	198

**Table 2:** Onset and duration of anesthesia.

Parameters	Group I (Mean ± SD)	Group II (Mean ± SD)	Statistical analysis
Onset (seconds)	162.92 ± 64.4	176.03 ± 58.2	*P* = 0.121
Duration of anesthesia (minutes)	151.01 ± 44.4	111.07 ± 24.9	*P* = 0.00*

**Table 3:** Depth Of anesthesia using the VAS.

VAS score	Group I	Group II	Statistical analysis
0	82 (82%)	81 (83%)	*P* = 0.134
1	18 (18%)	17 (17%)	*P* = 0.135

Total	100	98	

**Table 4:** Comparison of mean heart rate at various time intervals using Centbucridine and Lignocaine.

Heart rate	Centbucridine		Lignocaine	
Base line	75.50	*P* value	76.61	*P* value
10 min after LA	76.16 (±4.16)	0.00*	77.42 (±2.73)	0.00*
20 min after LA	75.52 (±4.26)	0.65	76.61 (±3.31)	X
30 min after LA	75.50 (±4.28)	X	76.61 (±3.31)	X
60 min after LA	75.50 (±4.28)	X	76.61 (±3.31)	X
